# Bioactive Quinic Acid Derivatives from *Ageratina adenophora*

**DOI:** 10.3390/molecules181114096

**Published:** 2013-11-14

**Authors:** Mei Zhang, Wan-Xue Liu, Meng-Fei Zheng, Qiao-Lin Xu, Fang-Hao Wan, Jing Wang, Ting Lei, Zhong-Yu Zhou, Jian-Wen Tan

**Affiliations:** 1 Key Laboratory of Plant Resources Conservation and Sustainable Utilization, South China Botanical Garden, Chinese Academy of Sciences, Guangzhou 510650, China; 2 State Key Laboratory for Biology of Plant Diseases and Insect Pests, Institute of Plant Protection, Chinese Academy of Agricultural Sciences, Beijing 100193, China; 3 University of Chinese Academy of Sciences, Beijing 100049, China; 4 Biotechnology Division, Guangdong Academy of Forestry, Guangzhou 510520, China

**Keywords:** *Ageratina adenophora*, qunic acid, antimicrobial activity, radical scavenging capacity, DPPH

## Abstract

A novel quinic acid derivative, 5-*O*-*trans*-*o*-coumaroylquinic acid methyl ester (**1**), together with three known ones, chlorogenic acid methyl ester (**2**), macranthoin F (**3**) and macranthoin G (**4**), were isolated from the aerial parts of the invasive plant *Ageratina adenophora* (Spreng.). The structure of new compound **1** was elucidated on the basis of extensive spectroscopic analysis, including 1D- and 2D-NMR techniques. Compounds **2**–**4** were isolated from plant *A. adenophora* for the first time. All the compounds showed *in vitro* antibacterial activity toward five assayed bacterial strains, especially **3** and **4**, which showed *in vitro* antibacterial activity against *Salmonella enterica* with MIC values of 7.4 and 14.7 μM, respectively. Compound **1** was further found to display *in vitro* anti-fungal activity against spore germination of *Magnaporthe grisea* with an IC_50_ value 542.3 µM. These four compounds were also tested for their antioxidant activity against DPPH (1,1-diphenyl-2-picrylhydrazyl) radical.

## 1. Introduction

*Ageratina adenophora* (Spreng.) King & Robinson, native to Mexico and Costa Rica, is a perennial, herbaceous invasive plant which has invaded around 30 countries in tropical and subtropical zones of the world [1,2]. This plant was first introduced to Yunnan Province of China in the 1940s, and by now it has rapidly spread across a large area of southwest China, including Yunnan, Guizhou, Guangxi, Sichuan, Chongqing and Xizang provinces [3]. The rapid spread of *A. adenophora* in China has caused serious economic losses to agriculture, forestry and livestock, and damaged the ecology and environment of China’s native habitat [4,5].

**Figure 1:**
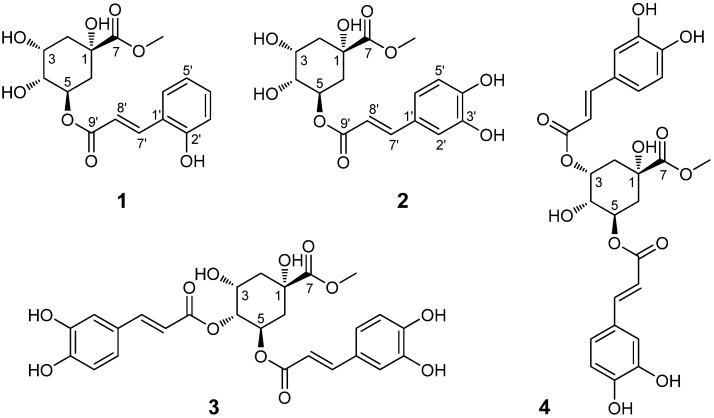
Chemical structures of compounds **1**−**4**.

*A. adenophora* is seldom attacked by bacteria, fungi and insects, suggesting that rich bioactive secondary metabolites that might be defense related, might exist in this plant. Previously, structurally diverse chemicals including (mono-, sesqui-, di-, and tri-) terpenoids, phenylpropanoids, flavonoids, coumarins, sterols and alkaloids were reported from this species [6,7,8], some of which were shown to possess allelopathic [9,10], phytotoxic [11] and antifeedant [12] activities. Our study reported herein has further led to the isolation of a novel compounds, 5-*O*-*o*-coumaroylquinic acid methyl ester (**1**), and three known quinic acid derivatives **2**–**4** from this species (Figure 1). We report the isolation and structural elucidation of these compounds, as well as their antimicrobial and DPPH radical scavenging activities.

## 2. Results and Discussion

**Figure 2:**
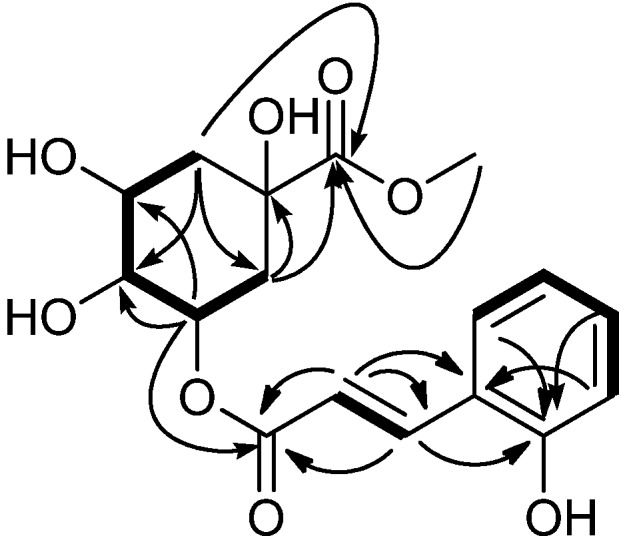
Key HMBC (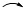) and COSY (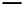) correlations of compound **1**.

Compound **1** was obtained as a yellowish gum. HR-ESI-MS (positive mode) showed a [M+Na]^+^ ion at *m/z* 375.1049, corresponding to the molecular formula C_17_H_20_O_8_ (calcd. for C_17_H_20_NaO_8_, 375.1056). IR absorptions at 3411 cm^−1^, 1733 cm^−1^ and 1654 cm^−1^, implied the existence of hydroxyl and carbonyl groups. In its ^13^C-NMR and DEPT spectra, the seventeen carbon signals of the molecule (1×C, 3×CH, 2×CH_2_, 1×CH_3_, two carbonyl group C-atoms and eight *sp^2^* C-atoms) could all be assigned (see Table 1). The presence of a quinic acid methyl ester moiety in the molecule was suggested by the presence of carbon signals at *δ*_C_ 75.8 (C), 38.0 (CH_2_), 70.3 (CH), 72.5 (CH), 72.1 (CH), 37.7 (CH_2_), 175.4 (C) and 53.0 (CH_3_), confirmed by the presence of proton signals at *δ*_H_ 2.19 (1H, Hα-2), 2.00 (1H, Hβ-2), 4.13 (1H, H-3), 3.74 (1H, H-4), 5.28 (1H, H-5), 2.19 (2H, H_2_-6) and 3.69 (3H, 7-OCH_3_) in its ^1^H-NMR spectrum. Coupled with ^1^H-^1^H COSY and HSQC spectral analysis, aromatic and olefinic proton signals of an *o*-coumaric acid (or 2-coumaric acid) moiety at *δ*_H_ 6.83 (1H, H-3'), 7.20 (1H, H-4'), 6.83 (1H, H-5'), 7.45 (1H, H-6'), and *δ*_H_ 7.91 (1H, H-7'), 6.58 (1H, H-8'), were all observed. Careful analysis of the ^1^H- and ^13^C-NMR spectra revealed that the NMR data of **1** were closely related to those of chlorogenic acid methyl ester [13], a known quinic acid derivative which was also obtained in the present study (compound **2**, see Table 1). The major difference was that the resonances for the substituted caffeoyl moiety in **2** were replaced by the signals for an *o*-coumaric acid moiety in **1**. These data indicated that **1** has the same quinic acid methyl ester moiety as that in **2** and further supported us to preliminarily establish the whole structure of **1** as 5-*O*-*trans*-*o*-coumaroylquinic acid methyl ester, of which the connectivity and the stereochemistry of the substituted *o*-coumaric acid moiety still needed to be determined. In the HMBC spectrum, the observation of ^1^H-^13^C long-range correlations of H-7' (*δ*_H_ 7.91) with C-2' (*δ*_C_ 158.4) and C-6' (*δ*_C_ 130.4), H-8' (*δ*_H_ 6.58) with C-1' (*δ*_C_ 122.5) and C-9' (*δ*_C_ 168.6) indicated the direct linkage of C-1' with C-7', and C-8' with C-9' (Figure 2).

**Table 1:** The ^1^H and ^13^C-NMR spectral data of **1** and **2** (*δ* in ppm and *J* in Hz).

Position	*δ*_C_ (1)	*δ*_H_ (1)	*δ*_C_ (2)	*δ*_H_ (2)
1	75.8		75.8	
2α	38.0	2.00 (dd, 13.6, 6.8)	38.0	1.99 (dd, 13.6, 6.8)
2β		2.19 (overlapped)		2.19 (overlapped)
3	70.3	4.13 (m)	70.3	4.13 (m)
4	72.5	3.74 (m)	72.5	3.72 (dd, 7.2, 3.2)
5	72.1	5.28 (m)	72.1	5.26 (m)
6α	37.7	2.18 (overlapped)	37.7	2.18 (overlapped)
6β		2.18 (overlapped)		2.18 (overlapped)
7	175.4		175.4	
7-OCH_3_	53.0	3.69 (s)	53.0	3.68 (s)
1'	122.5		127.6	
2'	158.4		115.1	7.03 (d, 2.0)
3'	117.0	6.83 (overlapped)	146.8	
4'	132.7	7.20 (td, 8.0, 1.2)	149.7	
5'	120.8	6.83 (overlapped)	116.5	6.77 (d, 8.0)
6'	130.4	7.45 (dd, 8.0, 1.2)	123.0	6.94 (dd, 8.0, 2.0)
7'	142.7	7.91 (d, 16.0)	147.2	7.51 (d, 16.0)
8'	118.4	6.58 (d, 16.0)	115.0	6.21 (d, 16.0)
9'	168.6		168.3	

Data were measured at 400 MHz for ^1^H and 100 MHz for ^13^C in CD_3_OD.

In the ^1^H-NMR spectrum, the presented coupling constants between H-7' and H-8' olefeinic protons (*J*_7',8'_ = 16.0 Hz) revealed that the double bond in the *o*-coumaric acid moiety was in the *E* geometry. The ester bond linkage between C-5 and C-9' was revealed by the observation of a significant HMBC correlation of *δ*_H_ 5.28 (H-5) with *δ*_C_ 168.6 (C-9'). In addition, the location of the hydroxyl group at C-2' was supported by HMBC correlations of H-4' (*δ*_H_ 7.20), H-6' (*δ*_H_ 7.45) and H-7' (*δ*_H_ 7.91) with C-2' (*δ*_C_ 158.4). The observation of NOE correlations between H-2β (*δ*_H_ 2.19), H-6β (*δ*_H_ 2.18) and H-4 (*δ*_H_ 3.74) in the NOESY spectrum, and the negative optical rotation value ([*α*] −22.2) indicated that the hydroxyl group at C-1 of **1** was an α- (axial) configuration [14]. Thus, **1** was elucidated as 5-*O*-*trans*-*o*-coumaroylquinic acid methyl ester as shown in Figure 1 and Figure 2. All the spectral data supported this structure.

The three known quinic acid derivatives were determined as chlorogenic acid methyl ester (**2**) [13], macranthoin F (**3**) and macranthoin G (**4**) [15], by interpretation of their spectroscopic data and comparison with literature values. They were isolated from *A. adenophora* for the first time.

These four compounds were tested for their *in vitro* antibacterial activities against five bacterial strains, including two Gram-positive (*Staphylococcus aureu* and *Bacillus thuringiensis*) and three Gram-negative (*Escherichia coli*, *Salmonella enterica* and *Shigella dysenteria*) bacterial species. The experimental results obtained from the bioassay (Table 2) revealed that **1**–**4** were all active compounds toward the five test bacterial strains, especially for **3** and **4**, which showed *in vitro* bacteriostatic activity against *S. enterica* with MIC values (7.4 and 14.7 μM, respectively) close to that of the positive control compound kanamycin (MIC 3.4 μM to *S. enterica*).

**Table 2:** MIC values of compounds **1**–**4** in μM against five bacterial strains.

Compounds	*Staphylococcus aureus*	*Bacillus thuringiensis*	*Escherichia coli*	*Salmonella enterica*	*Shigella dysenteriae*
**1**	88.8	88.8	88.8	88.8	177.6
**2**	84.8	84.8	84.8	84.8	169.8
**3**	29.4	59.0	59.0	14.7	117.9
**4**	59.0	59.0	59.0	7.4	117.9
KS	6.7	6.7	3.4	3.4	3.4

KS = Kanamycin sulfate.

These compounds were also tested for their antifungal activity against spore germination of the rice pathogenic fungus *Magnaporthe grisea* and their antioxidant activity against DPPH radical by using the bioassay methods as indicated in the Experimental section. Compound **1** was found to show *in vitro* anti-fungal activity against spore germination of *M. grisea* with IC_50_ 542.3 µM. Compounds **2** and **4** showed scavenging activity against DPPH radical, with SC_50_ values 212.2 and 150.2 µM, respectively, but they were much weaker than the positive control resveratrol (SC_50_ 42.1 µM).

Among these compounds, **1** is a novel chlorogenic acid derivative ester bond linked with an *o*-coumaric acid (2-coumaric acid). Generally, chlorogenic acid related compounds are formed by a quinic acid unit ester linked with one or more caffeoyl or *p*-coumaroyl unit(s) [16,17,18]. It is rather rare for this group of natural products to contain an *o*-coumaric acid unit in the structure.

*A. adenophora* is a well-known invasive plant which has spread rapidly and caused great economic loss in China. It has been suggested that allelopathy could be an important strategy for this plant to achieve its invasive success [19,20]. Recent study revealed that *o*-coumaric acid is phytotoxic and richly abundant in *A. adenophora*, which was suggested to be probably the most important allelochemical in this invasive species [11]. Taking the structural features into consideration, it is reasonable to predict that **1** might play a role in regulating the allelopathy of *A. adenophora* by functioning as a storage form of the strongly phytotoxic compound *o*-coumaric acid.

## 3. Experimental

### 3.1. General

Optical rotations were obtained on a Perkin-Elmer 341 polarimeter (Waltham, MaA, USA) with MeOH as solvent. UV spectra were recorded in MeOH on a Perkin-Elmer Lambda 35 UV-vis spectrophotometer. IR spectra (KBr) were recorded on a Bruker Tensor 27 spectrophotometer (Karlsruhe, German) in cm^−1^. ^1^H (400 MHz), ^13^C (100 MHz), and 2D NMR spectra were recorded in DMSO-*d*_6_ and CD_3_OD on a Bruker DRX-400 instrument with TMS as an internal standard. HR-ESI-MS data were obtained on a Waters Q-TOF Premier mass spectrometer (Milford, MA, USA). ESIMS were collected on an MDS SCIEX API 2000 LC/MS/MS instrument (Applied Biosystems, Inc., Forster, CA, USA). Preparative HPLC was conducted using a CXTH P3000 HPLC pump (Beijing Chuangxintongheng Science and Technology Co., Ltd, Beijing, China) and a UV3000 UV-vis Detector with a Fuji-C18 column (10 µm, Beijing Chuangxintongheng Science and Technology Co., Ltd). For column chromatography (CC), silica gel (200-300 mesh, Qingdao Marine Chemical Inc., Qingdao, China), YMC ODS-A (50 μm, YMC Co. Ltd., Kyoto, Japan) were used, and Sephadex LH-20 (Pharmacia Fine Chemical Co. Ltd., Uppsala, Sweden) were used. Fractions were monitored by TLC, and spots were visualized by heating the silica gel plates sprayed with 10% H_2_SO_4_ in ethanol.

### 3.2. Plant Materials

The aerial parts of *A. adenophora* were collected in a suburb of Kunming, Yunnan Province, China, in July 2009, and authenticated by Prof. Fu-Wu Xing, South China Botanical Garden, Chinese Academy of Sciences. A voucher specimen (No.20090702) was deposited at the Laboratory of Phytochemistry at the South China Botanical Garden, Chinese Academy of Sciences.

### 3.3. Extraction and Isolation

The air-dried aerial part material of *A. adenophora* (10 kg) were powdered and extracted three times with 95% EtOH in H_2_O (20 L × 3) at room temperature for 24 h each time. After removal of the ethanol (EtOH) *in vacuo*, the viscous concentrate was suspended in 10% ethanol in H_2_O (2.5 L) and then successively extracted with petroleum ether (3 × 3.0 L) and EtOAc (3 × 3.0 L). The EtOAc extraction solutions were then evaporated *in vacuo* to yield an oily EtOAc extract (80.0 g) which was subjected to silica gel column chromatography (CC) eluted with gradient of CHCl_3_/MeOH (95:5–60:40, v/v) to give fractions E_1_–E_14_. Fraction E_12_ (10.0 g), obtained by elution with CHCl_3_-MeOH (80:20, v/v), was subjected to silica gel CC eluted with CHCl_3_/MeOH (95:5–60:40, v/v) to obtain subfractions E_12-1_–E_12-6_. Fraction E_12-2_ (300 mg) was first purified by silica gel CC (petroleum-acetone 60:40, v/v) and further purified by HPLC using MeOH as mobile phase to afford compound **1** (*t_R_* = 66.1 min, 16.0 mg). Fraction E_12-3_ (270.0 mg)was first subjected to silica gel CC eluted with CHCl_3_/MeOH (20:1, v/v) and further purified by Sephadex LH-20 (MeOH) column chromatography to afford compound **3** (9.0 mg). Fraction E_12-4_ (21.3 g) was further applied to an ODS CC eluted with MeOH/H_2_O (30:70–60:40, v/v). The subfraction obtained by elution with MeOH/H_2_O 30:70 (v/v) was further subjected to Sephadex LH-20 CC eluted with pure MeOH to afford compound **2** (100.0 mg) and compound **4** (150.0 mg).

*5-O-trans-o-Coumaroylquinic acid methyl ester* (**1**). Yellowish gum; 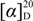 −22.2 (*c* 0.09, CH_3_OH); IR (KBr) *ν*_max_ 3,411, 1,733, 1,622, 1,259 cm^−1^; UV (MeOH) *λ*_max_ (log *ε*) nm: 212 (3.86), 276 (3.92); ESI-MS (+) *m/z*: 353 [M+H]^+^, 375 [M+Na]^+^; ESIMS (−) *m/z* 351 [M−H]^−^; HR-ESI-MS (pos.) *m/z* 375.1049 [M+Na]^+^ (calcd for C_17_H_20_NaO_8_, 375.1056); ^1^H-NMR (400 MHz, CD_3_OD) and ^13^C-NMR (100 MHz, CD_3_OD) data: see Table 1.

*Chlorogenic acid methyl ester* (**2**). Yellow solid; ESI-MS (+) *m/z*: 369 [M+H]^+^, 391 [M+Na]^+^; ESI-MS (−) *m/z* 367 [M−H]^−^; For ^1^H-NMR (400 MHz, CD_3_OD) and ^13^C-NMR (100 MHz, CD_3_OD) data, see Table 1.

### 3.4. Antibacterial Assay

The antibacterial activities of **1**–**4** were tested by using a microdilution method as reported in literature [21], with modification in determination of the minimum inhibitory concentration (MIC) values [22]. In the test, indicator solution (resazurin, 100 μg/mL, 100 μL) was first placed into each control wells (11th column) in 96-well microplates for the assay. Subsequently, indicator solution (100 μg/mL, 7.5 mL) was mixed with test organism (10^6^ cfu/mL, 5 mL) followed by transferring (100 μL, each) to growth control wells (12th column) and all test wells (1–10th column) in the 96-well microplates. Then, each of the sample solutions (1.0 mg/mL of test compounds in methanol, 100 μL) and positive control solution (1.0 mg/mL of kanamycin sulfate in methanol) as well as negative control sample (pure MeOH) were applied to the wells in the 1st column of the plates. In each test microplate, the four compound samples along with a positive control and a negative control samples were applied. Once all samples and controls were properly applied to the 1st column of wells in the microplates, half of the homogenized content (100 μL) from these wells was then transferred parallel to the 2nd column of wells, and each subsequent column of wells was treated similarly (doubling dilution) up to the 10th column, followed by discarding the last 100 μL aliquot. Finally, the plates were incubated at 37 °C for 5–6 h until the color of growth control wells change to pink. The lowest concentration for each test compound at which color change occurred was recorded as its primary MIC value. The averages of primary values from three individual tests were calculated and that was taken as the final MIC values for the test compounds [23]. Two Gram-(+) bacteria strains, *S. aureus* and *B. thuringiensis*, and three Gram-(−) bacterial species, *E. coli*, *S. enterica* and *S. dysenteria*, were used in the assay. MIC values for test compounds were displayed in Table 2.

### 3.5. Antifungal Assay

The inhibitory activities of test compounds against spore germination of *M. grisea* were tested by a microdilution assay. Briefly, the mixed solution containing fungal spore suspension solution (10^6^ spores/mL, 40 µL), test compound solution (5 µL) and 10% glucose solution (5 µL) were incubated on a concave glass at 28 °C in darkness for spore germination for 2.5 h. The germinated spores were then checked and recorded under a microscope. The solution concentrations of each test compound were set in the range of 2–200 μg/mL, and each of the test compounds were assayed in triplicate. A mixed solution for incubation without test compounds was used as negative control, and ketoconazole was used as a positive reference compound. The reported IC_50_ value represents the concentration of a test compound required to inhibit 50% of spore germination.

### 3.6. Determination of Antioxidant Activities

The antioxidant activities of test compounds were determined by the DPPH assay as previously described [24]. Briefly, the reaction mixture containing sample solution (20 μL) and DPPH (180 μL, 150 μM) in ethanol was placed in a 96-well microplate and incubated at 37 °C for 30 min. The absorbance was measured at 517 nm by a microplate reader. SC_50_ value represents the concentration of a compound to scavenge 50% of DPPH radicals. Resveratrol was used as positive control.

## 4. Conclusions

A new quinic acid derivative, 5-*O*-*trans*-*o*-coumaroylquinic acid (**1**), was isolated from the aerial parts of the invasive plant *A. adenophora* (Spreng.), along with three known ones **2**–**4**. The three known compounds were all found in this plant species for the first time. Compound **1** is a chlorogenic acid derivative ester bond linked with an *o*-coumaric acid unit in the molecule, which is rather rare in Nature. Antibacterial assays revealed that compounds **1**–**4** were all active toward the five assayed bacterial strains, especially compounds **2** and **4**, which showed *in vitro* antibacterial activity against *S. enterica* with MIC values (7.4 and 14.7 μM) very close to that of the positive control kanamycin (MIC 3.4 μM). Compound **1** was further found to display obvious *in vitro* anti-fungal activity against spore germination of *M. grisea*, with an IC_50_ of 542.3 µM. A DPPH radical scavenging assay demonstrated that **2** and **4** are slightly active, but much weaker than the famous polyphenol resveratrol.
